# Supplementary material for: Dissociating predictability, plausibility and possibility of sentence continuations in reading: evidence from late-positivity ERPs
Source: PeerJ. 2018 Oct 12;6:e5717. doi: 10.7717/peerj.5717 (PMC6187994; doi:10.7717/peerj.5717)
Supplement: Supplemental Information 3 [file peerj-06-5717-s003.pdf]

Table S1. German sentence pairs and critical nouns [EXP/USP/ANOM]

**EXP/USP/ANOM-Impos**	
1.	Alice brach sich ihr Bein im Wanderurlaub. Der Arzt röntge ihr Bein und legte es in einen [Gips/Rollstuhl/Vogel] für zehn Wochen.
2.	Annabell liebte die Show "Germany's Next Topmodel". Auch sie träumte von einem Lauf über einen [Laufsteg/Platz/Untergang] vor einem bedeutsamen Publikum.
3.	Lennard zog nach Hollywood und hielt sich mit Kellnern über Wasser. Eigentlich hoffte er aber auf eine Anstellung als ein [Schauspieler/DJ/Ei] bei einem angesagten Arbeitsgeber.
4.	Lisas Großmutter war sehr gebildet und weise. Bei Problemen hatte sie immer einen [Rat/Arzt/Mord] parat.
5.	Manche Dinge verlernt man nie. Dazu zählen zum Beispiel das Fahren eines [Fahrrads/Snowboards/Aliens] oder das Binden der Schürsenkel.
6.	Maria hasste es den Köder am Haken zu befestigen. Aber nur so fing sie einen [Fisch/Drachen/Rat] für das Abendessen.
7.	Marie war kurz überrascht, als sich die Augen ihres Bruders bei der Zubereitung des Gemüses mit Tränen füllten. Er schnitt allerdings nur eine [Zwiebel/Chilischote/Glocke] für das Abendessen.
8.	Marina pflanzte verschiedene Rosensorten in ihren Garten. Eine Sorte bezauberte mit einem [Duft/Geruch/Trainer], der unglaublich intensiv war.
9.	Marios Firma feierte diese Woche mit einem großen Programm ihr zwanzigjähriges Bestehen. Für das Bankett kaufte er sich extra einen [Anzug/Gürtel/Astronauten], um angemessen angezogen zu sein.
10.	Anne schrieb gerade ihre Masterarbeit und brauchte noch weitere Quellen für ihre Annahmen. Deshalb machte sie sich auf den Weg in eine [Bibliothek/Lehrbuchsammlung/Feder] für ihren Fachbereich.
11.	Marius war im Rahmen eines freiwilligen sozialen Jahres für zwölf Monate in Tansania. Bei seiner Rückkehr überraschten ihn seine Freunde mit einer [Party/Flagge/Entzündung] in seinem Elternhaus.
12.	Mark verließ sich immer auf seine Freunde, wenn er irgendwohin musste. Seit längerem sparte er nun aber Geld für den Kauf eines [Autos/Flugzeugs/Polizisten] in einem Monat.
13.	Martin war bescheiden, als er sagte, dass sein neues Haus eher groß war. Mit drei Flügeln und zehn Schlafzimmern war es eher eine [Villa/Insel/Autorin] als ein Haus.
14.	Meine Schwiegereltern haben einen wunderschönen Teich im Garten. Im Moment blüht in der Mitte eine [Seerose/Pflanze/Briefmarke] und kleine Fische schwimmen herum.
15.	Meistens bleibt Janine über Telefon oder Email mit anderen in Kontakt. Fast nie schreibt sie heutzutage einen [Brief/Text/Arzt] an ihre Freunde.
16.	Mike tanzte sehr gerne und wollte deshalb auf seiner Geburtstagsfeier gute Musik haben. Für die Party engagierte er einen [DJ/Elektriker/Strafzettel], der ein hervorragendes Soundsystem mitbrachte.
17.	Mona fuhr mit ihrem Fahrrad durch den Wald, als es anfing zu regnen. Um trocken zu bleiben, suchte sie Schutz unter einem [Baum/Stall/Alibi] nahe eines Hauses.
18.	Monika wusste nicht weiter und eröffnete Nick, dass sie ihn verlassen würde, wenn er sein Verhalten nicht ändere. In der Hoffnung ihre Beziehung zu retten, stellte sie ihm ein [Ultimatum/Angebot/Erdbeben] als letzten Ausweg.
19.	Moritz und seine Freunde hatten endlich ihre Klausurenphase beendet. Sie trafen sich in einer Bar und tranken ein [Bier/Wasser/Snowboard] als Belohnung.
20.	Astrid war sehr sensibel bezüglich ihres Gewichts. Als sie gefragt wurde, ob sie ein paar Pfunde zugenommen habe, empfand sie dies als eine [Beleidigung/Kränkung/Zeitung] von ihrem Kollegen.

21. Nach langer Überlegung fiel Karls und Kristins Entscheidung auf eine standesamtliche Trauung. Zu Beginn unterschätzten sie Aufwand und Planung einer [Hochzeit/Zeremonie/Nadel], die allen Interessen gerecht wird.
22. Natascha schlenderte durch die farbenfrohen Gärten. Obwohl es nicht erlaubt war, griff sie nach unten und pflückte eine [Blume/Orange/Einladung] von einem Busch.
23. Peter stand bei Morgendämmerung auf, fuhr den ganzen Tag Traktor und fütterte abends seine Kühe. An manchen Tagen wäre er aber lieber kein [Bauer/Erwachsener/Trick] sondern ein unbekümmertes Kind.
24. Robert lud Sina zu einem romantischen Abendessen ein. Im Kerzenschein tranken sie zum Hauptgericht einen [Wein/Tee/Babysitter] aus Italien.
25. Seit er klein war, hatte Steffen Interesse daran gehabt Filme zu machen. Und tatsächlich erfüllte sich vor einigen Jahren sein Traum und er wurde ein [Regisseur/Drehbuchautor/Rollstuhl] in einem großen Filmstudio.
26. Simon schaute jedes Spiel seiner Lieblingsmannschaft. Konnte er nicht zu den Spielen gehen, hisste er eine [Flagge/Leinwand/Party] in seinem Garten.
27. Simones Lieblingspullover hatte ein Loch am Ärmel. Sie kaufte Nähgarn und eine [Nadel/Schürze/Abiturientin], um es zu stopfen.
28. Susannes Schlafzimmer hat keine Rollladen zur Straße hin. Deshalb hätte sie gerne noch einen [Vorhang/Baum/Klempner] vor ihren Fenstern.
29. Susi musste einen Schal um ihre Hüfte binden, um ihre Jeans am Rutschen zu hindern. Beim Packen vergaß sie einen [Gürtel/Rock/DJ] zu Hause.
30. Sylvias Hund Rex war sehr selbstständig, putzte sich ständig und hielt gerne den ganzen Tag ein Nickerchen. Viele Personen fanden, er verhalte sich eher wie eine [Katze/Maus/Serviette] als wie ein Hund.
31. Thomas hatte ausnahmsweise im Halteverbot geparkt. Als er zurück zu seinem Auto kam, hatte er einen [Strafzettel/Brief/Archäologen] an seiner Scheibe.
32. Tilo spazierte mit seiner Tochter Maja am Strand. Zwischen zwei kleinen Felsen fand Maja eine [Muschel/Wasserschildkröte/Gunst] und freute sich.
33. Um Wortdefinitionen und die Rechtschreibung von Wörtern nachzuschauen, benutzte Jens ein Wörterbuch. Für Daten, historische Details und fachliches Wissen benutzte er ein [Lexikon/Handy/Fenster] als Referenz.
34. Viktoria war faul und der Meinung, dass sie keine anspruchslosen Arbeiten mehr machen müsse. Während alle anderen den ganzen Tag gearbeitet hatten, rührte sie keinen [Finger/Fuß/Unfall] auf der Arbeit.
35. Aus der Entfernung konnten wir nicht sagen, ob das Objekt in der Ferne ein Flugzeug war. Doch dann sahen wir den Flügelschlag und wussten, es war nur ein [Vogel/Bussard/Krankenwagen] in der Luft.
36. Violas Vater brachte ihr bei, wie man ein Omelett macht. Als erstes zeigte er ihr das Aufschlagen eines [Eies/Buches/Drachen] und das Anstellen des Herdes.
37. Vor einer Kneipe prügelten sich plötzlich zwei Männer. Zum Glück wartete in der Nähe ein [Polizist/Mann/Fisch] und eilte zur Hilfe.
38. Während des Probetrainings zeigte Raphael viel Können beim Halten der Bälle. Leider hatte das Fußballteam bereits einen [Torwart/Spieler/Strauß] für dieses Jahr.
39. Während seines Schulpraktikums grub Henri in Ruinen ein altes Grab aus. Nach dieser Erfahrung war sicher, er werde ein [Archäologe/Historiker/Tisch] nach der Schule.
40. Wenn kleine Kinder Insektenstiche haben, ziehen Eltern ihnen manchmal Socken über die Hände, um diese vom Kratzen abzuhalten. So verhindern sie eine Entzündung eines [Stichs/Fingers/Kochs] durch das Kratzen.

41. Zuerst dachten wir, der große Vogel in der Ferne sei ein Falke. Aber als wir den weißen Kopf sahen, wussten wir sofort, es war ein [Adler/Storch/Motor] am Himmel.
42. Zuerst wollte Julia das Porträt ihres Urgroßvaters in ihrer eigenen privaten Galerie aufhängen. Ihre Schwester überzeugte sie aber zu einer Ausstellung des wertvollen Kunstwerks in einem [Museum/Büro/Chemiker] in ihrer Heimatstadt.
43. Zum Nachtisch war das einzige Besteck, das an Tinas Platz lag, ein Löffel. Für den Kuchen fehlte eine [Gabel/Serviette/Garderobe] auf dem Tisch.
44. Aus gesundheitlichen Gründen musste der Bürgermeister in der Mitte seiner Amtszeit zurücktreten. Die Stadt veranstaltete deshalb erneut eine [Wahl/Bewerbung/Pflanze] in der Gemeinde.
45. Zwei Monate nach ihrem Umzug aß Dorothea ihr Essen immer noch von einem Umzugskarton. Ganz oben auf ihrer To-Do-Liste stand nun der Kauf eines [Tisches/Backofens/Regisseurs] für ihr Haus.
46. Beim Aufbau der Küche fehlten kurz vor Schluss wichtige Schrauben. Basti machte sich deshalb schnell auf den Weg zu einem [Baumarkt/Geschäft/Mikrofon] in der Nähe.
47. Alicias erster Klient war ein Misserfolg. Ihr zweiter Klient war jedoch ein [Erfolg/Witz/Baum] und gab ihr ihr Selbstbewusstsein zurück.
48. Bob behauptete aus dem Weltraum zu stammen. Allerdings glaubte ihm keiner, er sei ein [Alien/Engel/Pfiff] von einem anderen Planeten.
49. Charlotte wanderte durch eine schmale Schlucht umgeben von hohen Felswänden. Sie rief laut "Hallo" und hörte ein [Echo/Wort/Iglu] erklingen.
50. Claudius war sehr kreativ in der Küche und entwickelte ständig neue Rezepte. Vor einem Monat begann er deshalb eine Ausbildung zu einem [Koch/Bäcker/Gips], um seine Leidenschaft zum Beruf zu machen.
51. Das kleine Mädchen klebte einen gekringelten Schwanz an ihren pinken Schlafanzug und trug ein Paar rosane Ohren. Sie verkleidete sich als ein [Schwein/Kaninchen/Kompliment] an Karneval im Kindergarten.
52. Das Wichtigste an Nicoles Kostüm waren der Heiligenschein und die Flügel. Dieses Jahr verkleidete sie sich als ein [Engel/Alien/Echo] an Halloween.
53. Dennis träumt davon Planeten zu erkunden und ins Weltall zu fliegen. Wenn er erwachsen ist, wäre er sehr gerne ein [Astronaut/Forscher/Publikum] oder Wissenschaftler.
54. Als die Polizei Toni fragte, wo er in der Nacht des Mordes gewesen war, wusste er, dass er in Schwierigkeiten war. Er war alleine zu Hause und hatte deshalb kein [Alibi/Publikum/Dorf] für den Abend.
55. Der kleine Junge sagte seiner Großmutter auf Wiedersehen. Er schlang seine Arme um sie und gab ihr einen [Kuss/Strauß/Erwachsenen], bevor sie ging.
56. Der Kreideumriss des Körpers war immer noch auf dem Boden sichtbar. In der vorherigen Nacht beging jemand einen [Mord/Anschlag/Torwart] in einem Park.
57. Der Mann am Strand legte sein Board neben sich und zog seinen Neoprenanzug aus. Er war mit Sicherheit ein [Surfer/Tourist/Brand] oder ein Rettungsschwimmer.
58. Der Mann trug Khakihosen, ein grelles Hawaiihemd und eine Kamera um seinen Hals. Er war offensichtlich ein [Tourist/Regisseur/Fahrrad] in diesem Land.
59. Der neue Arbeitskollege erzählte uns, dass er in verschiedenen Ländern Medizin praktiziert hatte. Ohne einen Kittel sah er gar nicht aus wie ein [Arzt/Chemiker/Stich] in einem Krankenhaus.
60. Der Ort des Autounfalls sah entsetzlich aus. Sanitäter fuhren die Verletzten in einem [Krankenwagen/Taxi/Luftballon] zum Krankenhaus.
61. Der Rockstar rollte einen Ärmel seiner Lederjacke hoch und zeigte seinen Arm. Letzten Sommer stach ihm betrunken ein Freund ein [Tattoo/Schwein/Büro] in seinem Tattoo-Studio.

62. Der Tag war windig. Der Junge ging nach draußen und lenkte einen [Drachen/Helikopter/Brief] in der Nachbarschaft.
63. Die Ärzte würden es Monika nicht erlauben, so schnell nach der Operation selbstständig zu gehen. Ihre einzige Möglichkeit sich zu bewegen war ein [Rollstuhl/Gips/Eis] oder ein Paar Krücken.
64. Die Bäckerei akzeptierte keine Kreditkarten. Peter hatte kein Bargeld und schrieb dem Verkäufer deshalb einen [Scheck/Gutschein/Obstsalat] über den offenen Betrag.
65. Die Band entschied sich dagegen ein Live-Album aufzunehmen. Sie erhofften sich mehr Kontrolle über die Aufnahmen in einem [Studio/Raum/Elefanten] ohne Publikum.
66. Die Einwohnerzahl von Nordkirchen liegt bei etwa 9500 Menschen. Offiziell ist das eine Stadt, manche nennen es aber auch ein [Dorf/Fleckchen/Tattoo], weil es so klein ist.
67. Die Idee des Fliegens besteht bereits seit einigen tausend Jahren. Erst 1903 jedoch bauten die Gebrüder Wright ein [Flugzeug/Objekt/Kind], das flog.
68. Als Jakob von der Arbeit nach Hause kam, brachte er seiner Frau ein Dutzend Rosen mit. Sie kürzte die Stiele und stellte die Rosen in eine [Vase/Bibliothek/Katze] auf ein Regal.
69. Die Kinder fragten sich, ob das rostige, alte Auto in der Garage noch fahren würde. Als sie die Motorhaube öffneten, fehlten allerdings ein [Motor/Schlauch/Engel] und andere Teile.
70. Die Kinder liebten Halloween und gingen jedes Jahr von Haus zu Haus. Außerdem schnitzten sie immer einen [Kürbis/Tisch/Tee] als Deko.
71. Die Kinder spielten draußen in der Kälte und taten so, als wären sie Inuit. Die gewölbte Struktur aus Schnee, die sie bauten, sah allmählich aus wie ein [Iglu/Ei/Elektriker] und nahm den gesamten Vorgarten ein.
72. Die Kinder spielten zu nah am Haus Basketball. Ein Kind verfehlte den Korb und zerstörte durch seinen Wurf ein [Fenster/Schloss/Wort] der Garage.
73. Die kleine Theresa wäre gerne eine Prinzessin. Regelmäßig baut sie sich aus Pappkartons ein [Schloss/Dorf/Bier] in ihrem Zimmer.
74. Die Nachbarskatze verbringt fast den ganzen Tag in unserem Garten. Letzte Woche brachte sie uns eine [Maus/Feder/Antwort] ins Wohnzimmer.
75. Dieses Jahr hatte die Familie einen besonders schönen Tannenbaum gekauft. Auf die Spitze des Baumes befestigten die Kinder einen [Stern/Vogel/Forscher] als Krönung.
76. Als Marina die Papiere ausfüllte, musste sie den Namen eines Familienmitglieds oder eines engen Freundes angeben. Man würde diese Person kontaktieren im Falle eines [Notfalls/Erfolges/Architekten] bei der Arbeit.
77. Dieses Jahr kommt Julie in die Oberstufe des Gymnasiums. Nach drei Jahren ist sie dann eine [Abiturientin/Schulabgängerin/Blume], wenn sie besteht.
78. Eine Menschenmenge versammelte sich um die langbeinige Kreatur, deren Kopf im Sand steckte. Außer im Fernsehen hatten die meisten vorher noch keine Begegnung mit einem [Strauß/Laufvogel/Marathon] in natura.
79. Ellen wollte mehr Zitrusfrüchte essen, mochte aber keine Grapefruits. Deshalb schälte sie sich oft eine [Orange/Clementine/Gabel] als Snack.
80. Erika war besorgt in einer Region zu leben, unter der die Grenzen tektonischer Platten verliefen. Seit sie dorthin gezogen war, erlebte sie bereits ein [Erdbeben/Unglück/Lexikon] mittlerer Stärke.
81. Es gab schreckliche Probleme mit den elektrischen Leitungen des alten Hauses, das Sarah kürzlich gekauft hatte. Sie engagierte einen [Elektriker/Fachmann/Scheck], der die Leitungen reparierte.
82. Es gibt Altersbeschränkungen für bestimmte Filme. Filme ab zwölf Jahren sind zum Beispiel für jüngere Kinder erlaubt in Begleitung eines [Erwachsenen/Babysitters/Baumarkts] oder Erziehungsberechtigten.

83. Es gibt immer Autofahrer, die unvorsichtig fahren. Deshalb gerät fast jeder Autofahrer eines Tages in einen [Unfall/Krankenwagen/Stern] unabhängig von der eigenen Vorsicht.
84. Es ist immer eine Tragödie, wenn ein Kind seine Mutter oder seinen Vater verliert. Kinder haben große Schwierigkeiten beim Umgang mit dem Verlust eines [Elternteils/Mitbewohners/Witzes], wenn sie jung sind.
85. Als Timo zum ersten Mal den Pazifik sah, dachte er, dass dies ein See wäre. Sein Bruder lachte und erklärte ihm, dies vor ihm sei ein [Ozean/Teich/Tourist] und kein See.
86. Familie Schmidt fragte den Nachbarsjungen, ob dieser auf ihren Sohn Bobby aufpassen könnte, während sie außer Haus waren. Bobby war sauer, weil er der Ansicht war nun zu alt zu sein für einen [Babysitter/Beobachter/Wal] oder Aufpasser.
87. Frank wollte ein sehr modernes Haus gestalten. Für die Entwürfe suchte er Rat bei einem [Architekten/Klempner/Kleid], um wichtige Details zu besprechen.
88. Harald sah Rauch in der Ferne. Er nahm sofort sein Telefon und meldete einen [Brand/Qualm/Schauspieler] in der Nähe.
89. Heidi und Mike entfernten das Bett aus dem Zimmer und stellten stattdessen Schreibtisch, PC und Aktenschränke hinein. Sie verwandelten das zusätzliche Schlafzimmer in ein [Büro/Studio/Buch], um zu Hause arbeiten zu können.
90. Herr Breker hatte kurz vor Beginn der Saison eine Stelle bei einem anderen Verein angenommen. Bobbys Team hatte deswegen zunächst keinen [Trainer/Betreuer/Duft] und verlor mehrere Spiele.
91. Im Winter mit dem Schiff durch die Arktis zu reisen kann sehr tückisch sein. Es besteht immer die Gefahr vor einem Zusammenstoß mit einem [Eisberg/Wal/Namen] in der Dunkelheit.
92. Im Zoo liefen zwei Pfauen frei herum. Als Miriam sich umdrehte, fand sie eine [Feder/Muschel/Wahl] auf dem Boden.
93. In den meisten Lebensbereichen gibt es Regeln, die befolgt werden sollen. Aber für fast jede Regel gibt es unter bestimmten Umständen eine [Ausnahme/Ausrede/Muschel], die toleriert wird.
94. In den vergangenen Jahren hatte Jana ihre Steuern selbst verwaltet. Dieses Jahr beauftragte sie einen [Steuerberater/Onkel/Aufzug] mit der Arbeit.
95. Jan machte eine Reise nach Indien. Der Höhepunkt seines Aufenthalts war ein Ritt auf einem [Elefanten/Ochsen/Kuss] in einem Nationalpark.
96. Anna hatte gerade ihren zwölften Geburtstag gefeiert und bestand darauf, dass sie nun fast ein Teenager war. Allerdings verhielt sie sich noch wie ein [Kind/Küken/Flugzeug] die meiste Zeit über.
97. Jasmin ist eine begeisterte Läuferin und trainiert schon seit Wochen sehr intensiv. In einem Monat läuft sie einen [Marathon/Wettkampf/Jäger] in New York.
98. Jessy wollte keine Suppe auf ihrer neuen Bluse verschütten, während sie kochte. Um ihre Kleidung zu schützen, trug sie eine [Schürze/Jacke/Insel] beim Kochen.
99. Johanna war verzückt, als ihr erstes Buch für eine Veröffentlichung akzeptiert wurde. Endlich fühlte sie sich wie eine [Autorin/Abiturientin/Banane] gegenüber ihren Mitmenschen.
100. Judith kam zu spät zum Seminar. Ihr Fahrrad hatte einen [Platten/Notfall/Onkel] und sie musste den Bus nehmen.
101. Karen kam ursprünglich vom Festland und mochte den Gedanken nicht, von Wasser umgeben zu sein. Sie gewöhnte sich nie richtig an das Leben auf einer [Insel/Seerose/Schürze] so weit weg von Zuhause.
102. Karl kam auf dem Weg zur Arbeit an einem Kiosk vorbei und überflog die Schlagzeilen. Er bezahlte 50 Cent und kaufte eine [Zeitung/Briefmarke/Ausnahme] für später.
103. Karla studierte die Venusfliegenfalle für ihren Biologieunterricht. Obwohl diese Fleisch frisst, ist sie eine [Pflanze/Ausnahme/Nachricht] und kein Tier.

104. Kathrin war auf ihre erste Hochzeit eingeladen. Aufgeregt durchstöberte sie das Internet auf der Suche nach einem [Kleid/Hotel/Platten] für den Abend.

105. Lena war nicht laut genug, um von den Zuhörern gehört zu werden. Um ihre Stimme zu verstärken, benutzte sie deshalb ein [Mikrofon/Echo/Auto] auf der Bühne.

EXP/USP/ANOM-Pos

106. Luisas neues WG-Zimmer war sehr klein, hatte aber hohe Decken. Um Platz zu sparen, kaufte sie sich deshalb ein [Hochbett/Aufbewahrungssystem/Schwein] im Baumarkt.

107. Manuel ist ein begeisterter Skifahrer und fährt seit Jahren zum Urlaub in die Alpen. Dieses Jahr wollte er etwas Neues ausprobieren und fuhr zum ersten Mal ein [Snowboard/Auto/Studio] von einem Freund.

108. Marie musste dringend einen Brief abschicken. Sie fuhr zur Post und kaufte sich eine [Briefmarke/Einladung/Beleidigung] am Schalter.

109. Marleen war schüchtern und konnte nicht gut mit Lob umgehen. Sie war peinlich berührt durch ein [Kompliment/Tattoo/Bügeleisen] ihres Vorgesetzten.

110. Nachdem Anna zu Hause einen Film gesehen hatte, wollte sie nicht mehr mit ihren Freunden in eine Bar gehen. Um nicht gehen zu müssen, erfand sie eine [Ausrede/Idee/Vase] als Deckung.

111. Nina hatte vormittags Orangen, Äpfel, Weintrauben und eine Ananas auf dem Markt gekauft. Nach dem Mittagessen überraschte sie ihre Kinder mit einem [Obstsalat/Kürbis/Stall] als Dessert.

112. Saschas Familie besuchte die Reptilien-Ausstellung während ihres Besuchs in Florida. Der Tourguide erklärte ihnen den Unterschied zwischen einem Krokodil und einem [Alligator/Adler/Vorhang] anhand verschiedener Merkmale.

113. Auf der Bühne sah es so aus, als hätte der Zauberer den Körper der Frau in zwei Hälften gesägt. Wir mussten unseren Kindern erklären, dies sei nur ein [Trick/Unfall/Laufsteg] und nicht real.

114. Stefans Mutter war es Leid für ihren erwachsenen Sohn immer noch die Wäsche zu waschen. Zum Geburtstag schenkte sie ihm deshalb eine [Waschmaschine/Putzfrau/Orange], um seine Selbstständigkeit zu fördern.

115. Susanne wollte unbedingt zu Tims Hochzeit gehen. Sie kontrollierte ihre Post jeden Tag, aber erhielt keine [Einladung/Zeitung/Affäre] von ihm.

116. Ute gewann bei einem Gewinnspiel eine Reise nach Afrika. Höhepunkt dieser Tour war eine [Safari/Villa/Waschmaschine] in der Savanne.

117. Willi hoffte, dass er im Sommer in einem Fastfood-Restaurant arbeiten könne. Deshalb schickte er eine [Bewerbung/Antwort/Zwiebel] an verschiedene Restaurants.

118. Yannick ist ein begeisterter Leser. Zum Geburtstag schenkte ihm seine Schwester ein [Buch/Lexikon/Küken] über Afrika.

119. Barbara liebte es zu backen, konnte dies aber nicht in ihrer Wohnung tun. Zur Ausstattung gehörten leider kein [Backofen/Herd/Eisberg] und keine Mikrowelle.

120. Bei einem Spaziergang durch den Wald hörten wir plötzlich einen Schuss. Kurz darauf sahen wir einen [Jäger/Mord/Steuerberater] auf einem Feld.

121. Beim Abendessen verkündete Toms Schwester, dass sie ein Baby erwarte. Tom rief laut, er werde zum ersten Mal ein [Onkel/Pate/Gürtel] und klatschte in die Hände.

122. Bernd reinigte seine Wunde nicht gründlich genug. Deshalb bekam er kurze Zeit später eine [Entzündung/Krankenschwester/Seerose], die heilen musste.

123. Charlies Frau hatte keine Lust mehr, seine Hemden zu bügeln. Am folgenden Tag schenkte sie ihm ein [Bügeleisen/Kleid/Schloss] für seine Garderobe.

124. Dana genoss es lauthals zu singen, wenn sie alleine war. Aber sie hatte panische Angst vor einem Auftritt vor einem [Publikum/Mikrofon/Regenschirm] auf einem Konzert.

125. Daniel bedauerte, was er zu Bernadette gesagt hatte. Er schuldete ihr eine [Entschuldigung/Party/Sternschnuppe], das war sicher.

126. Der Biologe, den wir im Zoo getroffen haben, hatte spezialisiertes Wissen über Reptilien. Er erklärte uns den Unterschied zwischen einer Landschildkröte und einer [Wasserschildkröte/Maus/Bewerbung] anhand äußerer Merkmale.

127. Der Landwirt hielt die Kühe bei Tag und Nacht draußen auf der Weide. Er hätte sie gerne drinnen gehalten, hatte aber keinen [Stall/Architekten/Adler] auf seinem Bauernhof.

128. Der Stand hatte kein Helium mehr, als Frau Müller und ihre Tochter den Anfang der Schlange erreichten. Das Mädchen bekam keinen [Luftballon/Geschenkartikel/Bauern] und fing an zu weinen.

129. Als die Skifahrer das Donnern hörten und den rollenden Schnee sahen, rasten sie den Berg hinunter. Für diese Piste gab es vorab keine Warnung vor einer [Lawine/Tragödie/Bibliothek] diesen Ausmaßes.

130. Der Trainer belohnte seine Tiere mit ihrem Lieblingsessen. Der Otter erhielt einen Fisch und der Affe bekam eine [Banane/Möhre/Villa] als Belohnung.

131. Die Familie nahm wie jedes Jahr am Sankt-Martinsumzug teil. Dafür bastelten die Kinder in der Schule eine [Laterne/Sternschnuppe/Wohnung] aus Papier.

132. Die Gruppe hatte den ganzen Tag lang Brainstorming gemacht. Trotzdem fehlte ihr immer noch eine [Idee/Beleidigung/Möhre] für ihr Projekt.

133. Die Jungs wussten nicht, wie sie ihre Band nennen sollten. Auf Toms Vorschlag hin suchte jeder nach einem [Namen/Rat/Kürbis] und dann wurde abgestimmt.

134. Die Kirche im Dorf hat eine große Uhr an der Außenwand. Innerhalb des Turms, an einem langen Seil befestigt, befindet sich eine [Glocke/Schelle/Tragödie] aus Blei.

135. Die Schülerin hatte ihre Frage bereits fünf Mal gestellt. Sie wartete immer noch auf eine [Antwort/Nachricht/Hochzeit] des Lehrers.

136. Die Vorschülerin kombinierte die Buchstaben des Alphabets und formte das Wort "MAUS". Zum ersten Mal buchstabierte sie ein [Wort/Ergebnis/Unglück], das Sinn ergab.

137. Elena lag mit ihrem Freund auf einer Decke und blickte in den nächtlichen Himmel. Plötzlich sah sie zum ersten Mal in ihrem Leben eine [Sternschnuppe/Laterne/Entschuldigung] und wünschte sich etwas.

138. Endlich war der Sommer gekommen und Benjamin konnte wieder ins Freibad. Nachdem er lange im Wasser gespielt hatte, kaufte er sich ein [Eis/Bier/Museum] am Kiosk.

139. Es regnet oft in London. Unterwegs ist Nikolai deshalb immer mit einem [Regenschirm/Umhang/Finger] im Rucksack.

140. Es war schwierig den Gastprofessor zu verstehen. Wie viele ausländische Sprecher sprach er mit einem [Akzent/Lispeln/Mitbewohner] während seines Vortrags.

141. Frank hält sich selbst für einen Komiker. Trotzdem kennt er nicht einen [Witz/Schauspieler/Anzug] oder Sketch, über den sein Publikum lachen würde.

142. Für die Augen des Schneemanns verwendeten die Kinder zwei Stückchen Kohle. Für seine Nase benutzten sie eine [Möhre/Banane/Ausrede] aus dem Kühlschrank.

143. Heinrich war leicht klaustrophobisch. Er nahm lieber die Treppen statt eines [Aufzugs/Tunnels/Alligators] sofern möglich.

144. Andy suchte nach einem Ort, um den Stecker der neuen Lampe einzustöpseln. Er schaute überall, fand aber nirgendwo eine [Steckdose/Stelle/Krankenschwester] in seinem Schlafzimmer.

145. Ich rief bei Nora an, aber sie war nicht zu Hause. Netterweise hinterließ ihre Mutter eine [Nachricht/Entschuldigung/Wasserschildkröte] für sie.

146. Im neuen Zuhause seines Freundes legte Frederik seinen Mantel auf den Boden. Anscheinend hatte sein Freund noch keine [Garderobe/Kleiderstange/Flagge] in seiner Wohnung.

147. In Felicitas Badezimmer war ein Rohr gebrochen. Sie schaute im Telefonbuch nach einem [Klempner/Baumarkt/Herz] in ihrer Stadt.

148. In letzter Zeit hatte Frederike so viele Termine und Schwierigkeiten sich alle zu merken. Sie benötigte dringend einen [Kalender/Steuerberater/Teich], um sich besser zu organisieren.

149. In zwei Monaten würde Manuela ihre neue Stelle in einer anderen Stadt beginnen. Sie suchte immer noch nach einer [Wohnung/Waschmaschine/Maus] in der Nähe ihrer Arbeitsstelle.

150. Johannas Mann erzählte ihr, dass er jeden Abend lange im Büro bleiben musste. Allmählich hatte sie den Verdacht, er habe eine [Affäre/Liebschaft/Laterne] mit seiner Sekretärin.

FILL

151. Melanie war es Leid immer in die Uni zu fahren, um Dokumente auszudrucken. Zum Geburtstag wünschte sie sich deshalb einen [Drucker] und einen Vorratspack Patronen.

152. Der Lehrer wollte wissen, ob Hannes seine Hausaufgaben gemacht hatte. Er rief ihn an die Tafel und gab ihm eine [Aufgabe] aus dem Mathebuch.

153. Erika machte es sich auf einer Liege im Garten bequem. Um es sich noch gemütlicher zu machen, holte sie sich ein [Kissen] aus dem Wohnzimmer.

154. David hatte vergessen ein Auto zu reservieren. In der Autovermietung hatte er deshalb kein [Glück] und musste irgendeins nehmen.

155. Die Wahllokale waren nun geöffnet. Es war Zeit, dass die Wähler ihre Stimme gaben für eine [Partei] und einen Präsidenten.

156. Paula ging in den Wald, um Vögel zu beobachten. Leider vergaß sie ein [Fernglas] und konnte nur wenige Tiere sehen.

157. Gina erschrak kurz, als es plötzlich dunkel wurde. Doch vor die Sonne schob sich nur eine [Wolke], die kurze Zeit später weiter zog.

158. Seit einigen Wochen tauchen immer mehr Erdhäufchen auf unserem Rasen auf. Anscheinend lebt dort seit kurzem ein [Maulwurf] und treibt sein Unwesen.

159. Ingas Mittagessen hatte unerwartet Knoblauch enthalten. Da sie keine Zahnbürste dabei hatte, fragte sie ihren Kollegen nach einem [Kaugummi] oder einem Bonbon.

160. Marina war viel auf Reisen und erlebte fast jeden Tag etwas Neues. Um sich an alles zu erinnern, schrieb sie ein [Tagebuch] und klebte Fotos dazu.

161. Der Buchhalter realisierte, dass das Ergebnis seiner Rechnung nicht stimmen konnte. An einer Stelle in der Gleichung fand er einen [Fehler] und korrigierte ihn.

162. Dieter wollte seiner Schwester Blumen schenken, wusste aber, dass sie vergessen würde, diese zu gießen. Deshalb entschied er sich für einen [Kaktus] als Geschenk.

163. Dorotheas Ehemann war letzten Sommer verstorben. Seine Asche befindet sich auf dem Kaminsims in einer [Urne] im Wohnzimmer.

164. Leo fühlte, dass etwas über seinen Arm krabbelte, als er unter einen Busch griff. Er zog den Arm zurück und sah eine [Spinne] auf seiner Hand.

165. Nele hatte ihre Bewerbung an eine Firma geschickt. Nach Wochen des Wartens vereinbarte der Chef endlich ein [Vorstellungsgespräch] mit ihr.

166. Isabell hatte sich einen wunderschönen Mantel gekauft. Leider war dieser bei Regen unpraktisch, denn er hatte keine [Kapuze] und war aus Wolle.

167. Jakob wollte einige Bilderrahmen an der Wand befestigen, hatte aber nur Nägel. Deshalb fuhr er zum Baumarkt und kaufte einen [Hammer] und eine Wasserwaage.

168. Katharina wollte gerne Fische als Haustier halten. Deshalb kaufte sie sich zunächst ein [Aquarium] in einem Fachgeschäft.

169. Michael ist kein guter Freund von mir. Er ist eher ein [Bekannter] oder Kollege.

170. Jana wusste nicht, ob sie die Aufgabe ohne Hilfe lösen könnte. Sie startete trotzdem einen [Versuch] und fand die Lösung.
171. Felix' Lehrerin rief bei seinen Eltern an. Um über seine Noten zu sprechen, vereinbarte sie einen [Termin] mit ihnen.
172. Während ihrer Tour durch Kroatien wollten Hannes und Max der Natur möglichst nahe sein. Sie übernachteten deshalb in einem [Zelt] am Strand.
173. Silvia wachte morgens mit höllischen Zahnschmerzen auf. Doch sie konnte sich glücklich schätzen, ihr Mann war ein [Zahnarzt] und hatte seine Praxis in ihrem Haus.
174. Die Angestellte hatte noch eine halbe Stunde bis zum Feierabend. Sie räumte die Bücher zurück in ein [Regal] und räumte ihren Platz auf.
175. Mathilda beobachtete die Tauben in ihrem Garten. In der Tanne bauten sie ein [Nest], um Eier zu legen.
176. Meine Mutter hat vor kurzem das Onlineshopping für sich entdeckt. Fast jeden Tag kommt im Moment ein [Paket] mit der Post.
177. Beide Fußballteams hatten sich bereits auf dem Feld versammelt. Leider fehlte noch ein [Schiedsrichter], sodass das Spiel abgesagt werden musste.
178. Maike und Thorsten kennen sich schon eine ganze Weile, aber erst vor kurzem verliebten sich die beiden. Nun sind sie ein [Paar] und sehr glücklich.
179. Bereits Kinder wissen, was passiert, wenn es regnet und gleichzeitig die Sonne scheint. Dann sieht man gegenüber der Sonne einen [Regenbogen] am Himmel.
180. Letzten Monat wurde in Familie Müllers Haus eingebrochen. Um sich zu schützen, installierten sie jetzt eine [Alarmanlage] im Haus.
181. Am letzten Wochenende wurde in einen Supermarkt eingebrochen. Aus der Bevölkerung kam bis jetzt jedoch noch kein [Hinweis] bezüglich des Täters.
182. Wir waren überrascht, dass das Treffen nur so kurz war. Es dauerte nicht einmal eine [Stunde] und alles war besprochen.
183. Es war bitterkalt draußen und es wurde allmählich dunkel. Der Obdachlose suchte deshalb verzweifelt nach einem [Unterschlupf] für die Nacht.
184. Nach der Scheidung standen die Eheleute wegen eines Sorgerechtstreits vor Gericht. Letztendlich fanden sie dann aber doch eine [Lösung] bezüglich ihrer Kinder.
185. In unserer Nachbarschaft haben wir gestern einen Hund gefunden. Hoffentlich hat er einen [Besitzer] und ist kein Streuner.
186. Von seinem letzten Gehalt kaufte sich Igor einen neuen Computer, eine Maus und eine Tastatur. Jetzt fehlte nur noch ein [Bildschirm] und er war ausgerüstet.
187. Die Tochter von Carla und Bernhard sollte nun getauft werden. Am Nachmittag versammelte sich die gesamte Familie in einer [Kirche] und feierte die Taufe.
188. Ich wusste nicht, ob er die Wahrheit erzählte oder log. Aber dann zögerte er für einen [Moment] und ich wusste es.
189. In der Schule hatte Paul immer das Gefühl nicht richtig dazuzugehören. Er bezeichnete sich selbst als einen [Außenseiter] in seiner Klasse.
190. Mona und Rike wollten zu gerne mal wieder einen Film auf großer Leinwand sehen. Leider gab es in ihrer Stadt kein [Kino], sodass sie nur eine DVD schauten.
191. Vor Aufregung hatte die Studentin die ganze Nacht nicht geschlafen. Vor ihrer Prüfung in 30 Minuten benötigte sie noch dringend einen [Kaffee] oder eine Cola.
192. Die Kinder wollten gerne wissen, wo verschiedene Länder liegen. Ihr Vater holte deshalb einen [Atlas] aus dem Regal.

193. Der Mann im Anzug war nicht der, für den er sich ausgab. Wir warfen ihm vor, er wäre ein [Betrüger], und er floh.

194. Annika ist im Moment auf Wolke Sieben. Sie hat endlich wieder einen [Freund], in den sie sehr verliebt ist.

195. Die Eheleute waren nun schon sehr alt und der Meinung, es wäre Zeit ihr Erbe zu verteilen. Sie gingen zum Notar und formulierten ein [Testament] für ihre Kinder.

196. Heutzutage wollen die meisten nach der Schule studieren. Ich entschied mich jedoch für eine [Ausbildung] zum Bankkaufmann.

197. In der Grundschule mussten alle Kinder für den Chor vorsingen. Ich fand das sehr schrecklich, bin ich doch einfach kein [Sänger] sondern ein Sportler.

198. Gisela hatte für fünf Personen gedeckt. Unerwartet brachte ihre Freundin zum Abendessen einen Bekannten mit und es fehlte ein [Gedeck] auf dem Tisch.

199. Alina wollte die Chipstüte öffnen. In der Küche suchte sie deshalb nach einer [Schere], fand aber keine.

200. Walter wollte sein eigenes Unternehmen gründen. Bei der Bank beantragte er deshalb einen [Kredit], um Startkapital zu haben.
