# Supplementary material for: Dissociating predictability, plausibility and possibility of sentence continuations in reading: evidence from late-positivity ERPs
Source: PeerJ. 2018 Oct 12;6:e5717. doi: 10.7717/peerj.5717 (PMC6187994; doi:10.7717/peerj.5717)
Supplement: Supplemental Information 4 [file peerj-06-5717-s004.pdf]

Table S2. Interpolated electrodes

Participant	Interpolated electrode(s)
2	P4
16	T8
18	C3
20	O2
21	P3
32	T7, T8
