# Supplementary material for: Dissociating predictability, plausibility and possibility of sentence continuations in reading: evidence from late-positivity ERPs
Source: PeerJ. 2018 Oct 12;6:e5717. doi: 10.7717/peerj.5717 (PMC6187994; doi:10.7717/peerj.5717)
Supplement: Supplemental Information 5 [file peerj-06-5717-s005.pdf]

Table S3. One-sample t-tests of linear regression t-values

	t(31)	*p*	95% confidence interval
Plausibility	-1.73	.094	[-0.77; 0.06]
Possibility	0.60	.553	[-0.28; 0.51]
Word length	3.25	.003*	[0.19; 0.81]
Word frequency	-0.54	.595	[-0.45; 0.26]
Ortographic neighborhood size	1.62	.115	[-0.08; 0.67]

* Significant after Bonferroni adjustment ($\alpha_{boncor} < 0.01$)
