# Supplementary material for: Dissociating predictability, plausibility and possibility of sentence continuations in reading: evidence from late-positivity ERPs
Source: PeerJ. 2018 Oct 12;6:e5717. doi: 10.7717/peerj.5717 (PMC6187994; doi:10.7717/peerj.5717)
Supplement: Supplemental Information 6 [file peerj-06-5717-s006.pdf]

Correlation analysis of plausibility and possibility ratings

Table S4. Correlation between plausibility and possibility ratings

Condition	r	n	t	p
EXP	0.73	150	13.11	< .001
USP	0.75	150	13.71	< .001
ANOM	0.40	150	5.33	< .001
- ANOM-Pos	0.28	45	1.88	.068
- ANOM-Impos	0.21	105	2.16	.033

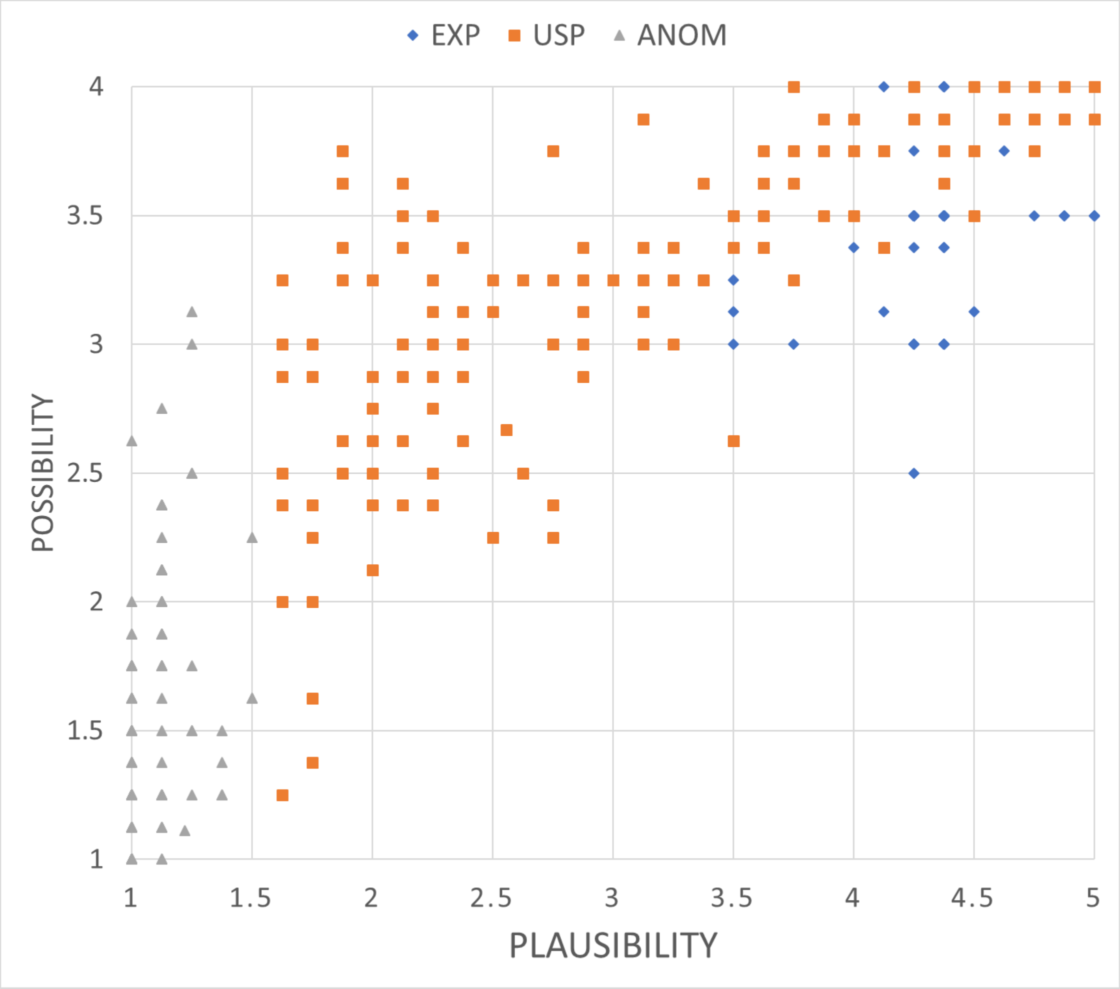

Figure S1. Distribution of items as a function of plausibility and possibility ratings.
