# Supplementary material for: Dissociating predictability, plausibility and possibility of sentence continuations in reading: evidence from late-positivity ERPs
Source: PeerJ. 2018 Oct 12;6:e5717. doi: 10.7717/peerj.5717 (PMC6187994; doi:10.7717/peerj.5717)
Supplement: Supplemental Information 7 [file peerj-06-5717-s007.docx]

Lateralization of anterior positivity

To confirm the left lateralization of the anterior positivity, we extended the corresponding ANOVA by the factor hemisphere (left, right), and included equivalent right hemisphere electrodes (Fp2, F4, F8, Fc6, and T8) while excluding midline electrodes Fz and Fpz. We found a main effect of hemisphere [*F*(1, 31) = 25.32, *p* < .001, η_p_² = 0.06], and a main effect of condition [*F*(2, 62) = 3.52, *p* = 0.036], as well as an interaction effect [*F*(2, 62) = 4.30, *p* = 0.018]. Pairwise t-tests are summarized in the Table S5 and values are displayed in Fig. S2.

| **Table S5**. Pairwise t-tests between noun types for both hemispheres | | |
| --- | --- | --- |
| Pairwise t-tests | Left hemisphere | Right hemisphere |
| EXP vs. USP | *t*(31) = -3.86, *p* < .001 | *t*(31) = -2.06, *p* = .048 |
| EXP vs. ANOM | *t*(31) = -2.00, *p* = .055 | *t*(31) = -0.59, *p* = .560 |
| USP vs. ANOM | *t*(31) = 1.28, *p* = .210 | *t*(31) = 0.84, *p* = .410 |
| *Uncorrected p-values* | | |


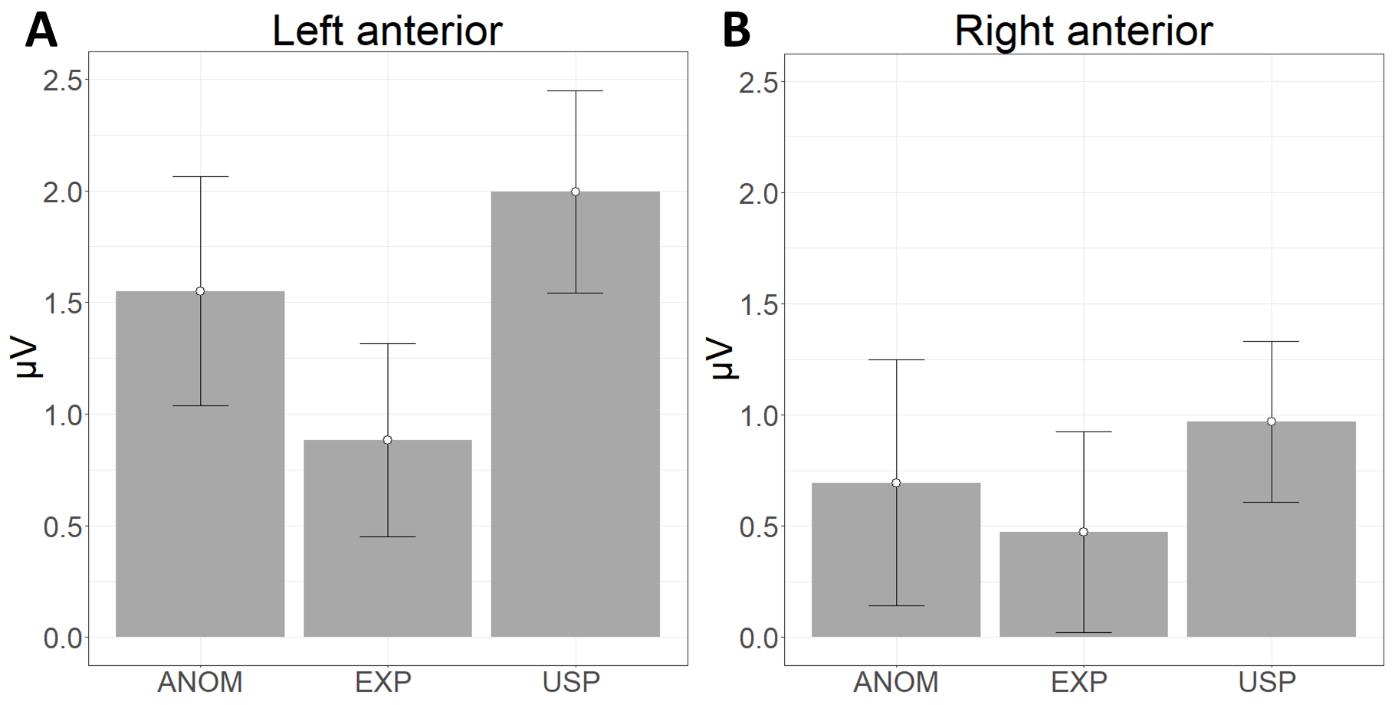


**Figure S2**. Mean amplitudes for 600 to 1000 ms and the three noun conditions (EXP, USP, ANOM) for left anterior (A) and right anterior (B) scalp channels. Error bars represent 95% confidence intervals (values are adjusted using method from Morey (2008)).

**Reference**

**Morey RD. 2008.** Confidence intervals from normalized data: a correction to Cousineau (2005). *Tutorials in Quantitative Methods for Psychology* **4(2)**:61-64 DOI 10.20982/tqmp.04.2.p061
